# Sublethal biochemical, behavioral, and physiological toxicity of extremely low dose of bendiocarb insecticide in *Periplaneta americana* (Blattodea: Blattidae)

**DOI:** 10.1007/s11356-023-25602-8

**Published:** 2023-02-06

**Authors:** Milena Jankowska, Bartosz Augustyn, Justyna Maliszewska, Barbara Przeździecka, Dawid Kubiak, Olga Chełchowska, Jagoda Kaczorek, Dominik Knop, Kornelia Krajnik, Hanna Kletkiewicz, Jacek Kęsy, Justyna Rogalska, Maria Stankiewicz

**Affiliations:** 1 Department of Animal Physiology and Neurobiology, Faculty of Biological and Veterinary Sciences, Nicolaus Copernicus University, Lwowska 1, 87-100, Torun, Poland; 2 Department of Cellular and Molecular Biology, Faculty of Biological and Veterinary Sciences, Nicolaus Copernicus University, Lwowska 1, Torun, Poland; 3 Department of Plant Physiology and Biotechnology, Faculty of Biological and Veterinary Sciences, Nicolaus Copernicus University, Lwowska 1, 87-100, Torun, Poland

**Keywords:** Bendiocarb, Trace amounts of insecticides, Health risk, Cockroach, Octopamine, Stress, Behavior

## Abstract

Insecticides are dedicated to impair the insect organisms, but also have an impact on other, non-target organisms, including humans. In this way, they became important risk factor for disturbance of physiological homeostasis and can be involved in the development of diseases or in deterioration of existing conditions. The influence of sublethal doses of various insecticides on vertebrates’ and invertebrates’ organisms has been previously observed. In this paper, we have evaluated the impact of exposure to extremely low dose of neurotoxin, bendiocarb (0.1 nM), a commonly used carbamate insecticide on a model organism in neurobiology—*Periplaneta americana*. The assessment was performed on all levels of animal organism from molecular (oxidative stress parameters: phosphorylation level of proteins, cAMP level, protein kinase A and C levels, and octopamine) to physiological (heart beat and gas exchange tests) and behavioral (motor skills assay, grooming test). Exposure to such a low level of bendiocarb did not cause direct paralysis of insects, but changed their grooming behavior, decreased heart rate, and increased gas exchange. We also observed the increased parameters of oxidative stress as well as stressogenic response to 0.1 nM bendiocarb exposure. Exposure to a trace amount of bendiocarb also increased sensitivity to effective doses of the same insecticide, thus acts as preconditioning. These results force us to reconsider the possible risk from frequent/continuous exposure to traces of pesticide residues in the environment to human health.

**Supplementary Information:**

The online version contains supplementary material available at 10.1007/s11356-023-25602-8.

## Introduction

Pest management is based on chemical insecticides that act mainly as neurotoxic agents. Their extensive use leads to concerns about the impact on the environment and non-target organisms. It was estimated that only 1% of released insecticides achieve their targets and 99% of them end up in the environment as pollutants (Ansari et al. 2014). Insecticide residues can be found not only in the soil (Silva et al. 2019) and water (Saeid et al. 2011) but also in fruits (Rodrigues et al. 2011), fishes (Chang et al. 2020), and plant pollen and nectar (Casado et al. 2019), as well as in arctic snow, where no pesticide had been ever applied (Pimentel 1995; Weber et al. 2010).

One of the broadly used insecticides is bendiocarb; it belongs to the class of carbamate insecticides. Carbamates inhibit an enzyme acetylcholinesterase (AChE), which leads to abnormal activity of nervous system and paralysis. Carbamates are one of the most commonly used insecticides in agriculture and other industries (Grupta and Milatovic 2018). The consequence of extensive use of bendiocarb is the accumulation of its residues in the environment (Gazsi et al. 2021). Bendiocarb was detected in surface waters in concentration range of µg/L in Japan (Tatarazako and Iguchi 2012) and Saudi Arabia (Saeid et al. 2011). Analysis of half-life of bendiocarb in the field revealed that its residues can be detected for 6 months after application (Maharaj et al. 2004).

The accumulation of insecticide residues has become a chemical stressor for both invertebrates and vertebrates (Del Prado-Lu 2015; Relyea 2005), causing the decline in species biodiversity (Sánchez-Bayo and Wyckhuys 2019). Sublethal doses of insecticides were shown to alter physiological parameters, such as activity of detoxifying enzymes in *Plutella xylostella* (LC_5_-LC_30_) (Chenyu and Xiaoming 2020) as well as behavior and oxidative stress markers in *Tubifex tubifex* (10% of LC_50_) (Chatterjee et al. 2021). Moreover, LC_20_ of sulfoxaflor showed transgenerational effect and enhanced the reproduction and survival rate of *Aphis gossypii* progeny (Shang et al. 2021). Exposure to carbamates in concentrations lower than lethal disturbs pupation and circadian rhythms (Sanil and Shetty 2012), growth rate, and whole-body protein content (Mosleh et al. 2003), and also alters metabolite content and behavior (Dewer et al. 2016) in various organisms. It was demonstrated that exposure to LD_20_ imidacloprid changed the expression pattern of genes important in nervous system functioning and detoxification enzymes in *Aphidius gifuensis* (Kang et al. 2018). Still, the tested concentrations of insecticides were much higher than detected residues found in environment. There is an abundant amount of data confirming the effects of high doses (lethal) of insecticides. However, the impact of trace amounts of insecticides needs evaluation, as they can be harmful to non-target organisms. In the presented study, we aim to explore the impact of extremely low dose of insecticide bendiocarb (22.3 ng/L) on a model organism in neurobiological studies—*Periplaneta americana*—in order to understand how residues of carbamates present in the environment can impact the insect organism. The detailed questions we asked in this paper were: how do trace amounts of bendiocarb influence organism 1) physiology, 2) stress response and 3) effectiveness of higher doses of insecticide.

## Materials and methods

### Insects

All experiments were performed on adult male American cockroach, *Periplaneta americana*. Insects were obtained from our own colony, reared in 29 ± 1 °C, and provided with oat flakes and dog chaw with access to water ad libitum. For the experiment, insects were randomly captured and moved to experimental conditions 24 h before experiment to allow them to adapt to the new conditions.

### First set of experiments—does exposure to 0.1 nM bendiocarb change the behavior of insects?

#### Insects’ exposure to bendiocarb

Bendiocarb (commercial product Ficam 80 WP; Bayer, Poland) was dissolved in 96% ethanol to the concentration of 10 mM. Serial dilutions of 100 µM, 1 µM, 10 nM, and 0.1 nM were then made in water. A concentration of 0.1 nM bendiocarb corresponds to 22.3 ng/L and is almost 1 million times lower than LD50 value (Jankowska et al. 2019a). Two groups of insects were distinguished: insects exposed to bendiocarb (0.1 nM Bend) and control insects (Ctr). The procedure of exposure to bendiocarb was performed as follows: 5 insects were placed in a round glass chamber (12 cm × 10 cm). Using glass atomizer, insects were sprayed with 1 mL of 0.1 nM bendiocarb solution. After 1 h of treatment, cockroaches were tested in experiments described further. All tests were performed in 22 ± 1 °C. The control group was treated with the same amount of water 1 h before the tests. Flowchart of experiments is presented in supplementary materials on Fig. SI1A.

#### Locomotor activity assessment

To evaluate how 0.1 nM bendiocarb solution affects the behavior of cockroaches, we performed a locomotor activity test. For a single trial, five cockroaches were placed in the 50-cm glass round arena where they were able to move freely. Walls of the arena were covered by paraffin, to prevent cockroaches from escape. After 5 min of adaptation, insects’ movement was recorded by video-camera and the output files were subsequently processed with the idTracker software (Stoelting, CO, USA) for 10 min. Data on the trajectory movement of single cockroach was analyzed using ad hoc scripts developed with MATLAB (MathWorks, Massachusetts, USA). Three parameters were analyzed: travelled distance, time spent on peripheral area (determined as 10% of the outer diameter of the arena), and time spent in immobility (counted if cockroach stopped for time longer than 0.5 s).

#### Grooming behavior assessment

The changes in grooming behavior in cockroaches are a suitable indicator of the presence of a chemical stressor in the environment (Weisel-Eichler et al. 1995). The cockroaches were moved to a clear container and grooming behavior was evaluated. The time of grooming of legs and antennas was counted with electrical stopwatch over 30-min period. Exposure and grooming evaluation were performed in constant temperature of 29 ± 1 °C, using thermostat and red-lamp heater.

#### Measurements of gas exchange (CO_2_ release)

Individuals were weighed prior to each experiment using analytical balance (Radwag AS 110/C/2). Recording of CO_2_ output for each individual was done using flow-through respirometry with Qubit Systems (Kingston, ON, Canada) data acquisition software controlling a 4-channel gas switcher and logging data from an infrared CO_2_ analyzer. A total of four channels were connected to the gas switcher: one baseline (empty) and three experimental (each containing an individual insect). Measurements were conducted in 30-mL chambers with a flow rate of 100 mL/min (time constant for volume exchange = 18 s). Gas controller and monitor were used to adjust the flow rate. Room air was pumped through the system with high vacuum-pump and column filled with magnesium perchlorate (Sigma-Aldrich, Poland) to be scrubbed of water. After leaving the experimental chamber, the air flew to the CO_2_ analyzer. The measurement of the CO_2_ exchange lasted 60 min. During a recording, three 5-min baselines (readings from an empty chamber) were determined, after each three experimental chamber readings. Baseline values were applied to provide accurate zero values. CO_2_ recordings were processed by Qubit Systems data analysis software. Software recorded CO_2_ levels in ppm and zeroed obtained values using baseline readings.

### Second set of experiments—does exposure to 0.1 nM bendiocarb change the oxidative status in insects?

#### Evaluation of oxidative stress markers

In this set of experiments, cockroaches were divided into two groups: one exposed to 0.1 nM bendiocarb and the control one. Flowchart of experiments is presented in supplementary materials on Fig. SI1A. The procedure of exposure is in accordance with the “Insects’ exposure to bendiocarb” section. Then, whole-body homogenates of control and exposed to bendiocarb cockroaches were prepared using a glass Potter homogenizer (Kleinfeld Labortechnik, Gehrden, Germany) in ice-cold phosphate buffer, pH 7.2. Samples were centrifuged at 12,000 g for 10 min at 4 °C. Supernatants were used for the determination of thiobarbituric acid reactive substances (TBARS) assay, reduced glutathione (GSH) concentrations, and catalase activity. For catalase activity measurements, supernatants were diluted 1:50 with 50 mM phosphate buffer (pH 7.0).

#### TBARS assay

TBARS assay quantifies oxidative stress by measuring the level of lipid peroxidation caused by reactive oxygen species. In this assay, thiobarbituric reaction product, malondialdehyde, was assessed spectrophotometrically according to the method of Buege and Aust (1978), modified by Cheeseman and Slater (1993). The samples were incubated with 15% trichloroacetic acid (TCA) and 0.37% thiobarbituric acid in boiling water bath for 20 min. Butylated hydroxytoluene in ethanol was added to the mixture to prevent from artefactual lipid peroxidation during the boiling step. After incubation, samples were centrifuged (15 min, 12,000 g) and the absorbance of supernatant was measured at 535 nm. MDA concentrations were calculated using molar extinction coefficient (156 mM^−1^L^−1^ cm^−1^).

#### Reduced glutathione (GSH) assay

Reduced GSH concentration was determined with the modified Ellman method (Ellman 1959). Supernatants from whole-body homogenates were mixed with 10% TCA and 10 mM EDTA and were centrifuged for 10 min at 10,000 g. The supernatant obtained from centrifugation was added to 2.3 mL of deionized water, 100 mL of 0.3 M EDTA, 300 mL of 0.32 M tris(hydroxymethyl)aminomethane, and 100 mL of 6 mM 5,5′-dithiobis-2-nitrobenzoic acid. Samples were maintained at 10 °C for 10 min and then the absorbance at 412 nm was measured.

#### Catalase activity measurements

Catalase activity (CAT) was quantified using modified method of Orta-Zavalza et al. (2014), based on the decomposition rate of hydrogen peroxide. The diluted sample was added to phosphate buffer and 30 mM H_2_O_2_. The breakdown of H_2_O_2_ was immediately measured for 3 min, at 1-min intervals, at 240 nm. CAT activity was determined using molar extinction coefficient for H_2_O_2_ at 240 nm (34.99 M^−1^ cm^−1^). One unit of CAT activity was defined as the amount that decomposes 1 µmol H_2_O_2_/min at 25 °C. Bradford method was used to determine the protein concentration in the samples (Bradford 1976). Bovine serum albumin was used to construct the calibration curve.

### Third set of experiments—does 0.1 nM bendiocarb affect the octopaminergic pathway?

#### Heartbeat measurements

In this experiment, insects were divided into three groups: control (Ctr), exposed to 0.1 nM of bendiocarb (0.1 nM Bend), and administrated with 0.1 mM octopamine (Oct). Flowchart of experiments is presented in supplementary materials on Fig. SI1C. Insects were deprived of wings and legs and were mounted to the cork platform by entomological needles, dorsal side down. Using microscissors, the abdomen of the cockroach was deprived of cuticle and digestive system was carefully moved aside, exposing the heart system. Preparation was kept wet with physiological saline (in mM: NaCl, 210; KCl, 3.1; CaCl_2_, 5; MgCl_2_, 5.4; and Hepes, 5; the pH = 7.2 was adjusted with NaOH) and was left for 10 min to accommodate. Then, the recording of the heartbeat was started under a stereoscopic microscope (Deltha Optical SZ-430 T) and camera (MEM1300). First, 5 min of basic heart activity was recorded; then, the thorax of the cockroach was sprayed with water (control) or 0.1 nM bendiocarb. In octopamine group, preparation was kept wet using 0.1 mM solution of octopamine in physiological saline (( ±)-octopamine hydrochloride, analytical standard (Sigma-Aldrich, Poland)); insects were sprayed with water as in the control group. Recordings were then continued for 30 min and the number of heartbeats per minute was counted by the experimenter.

#### Protein phosphorylation assay

In the following experiment, cockroaches were divided into 2 groups: control (Ctr) and exposed to 0.1 nM of bendiocarb (0.1 nM Bend). Flowchart of experiments is presented in supplementary materials on Fig. SI1A. Insects were exposed to bendiocarb as described in the “Insects’ exposure to bendiocarb” section. In each group, six abdominal and three thoracic ganglions were isolated from 3 exposed cockroaches and pulled together for each trial. Ganglions were placed in 1 mL of physiological saline and kept on ice until all ganglions were collected, and then, 2 mg of collagenase (from *Clostridium histolyticum* Type IA (Sigma-Aldrich, Poland)) was added and incubated for 30 min at 31 °C. Next, ganglions were gently washed with 1 mL of physiological saline. Tissues were homogenized using glass Pasteur pipettes. Phosphorylation level was determined using pIMAGO Phosphoprotein detection system (Tymora Analytical, USA), according to the producer’s instructions. Shortly, homogenate proteins were bounded to the 96-well plate, and after washing, plates were incubated with pIMAGO reagent, and afterwards with avidin-HRP conjugates. The level of phosphorylation was determined colorimetrically, reading the absorbance at 415 nm, using plate reader (BioTek, Epoch, Winooski, VT, USA). Phosphoprotein served as a positive control and albumin standard (Sigma-Aldrich, Poland) served as a negative control.

#### cAMP assay

Ganglions from two groups of cockroaches (control and exposed to 0.1 nM bendiocarb) were prepared as in the “Protein phosphorylation assay” section. Initially digested ganglions were moved to 0.1 M HCl and after that were homogenized using glass Pasteur pipettes. Samples were centrifuged (14,000 g, 5 min) and the supernatant was collected. Then, the level of cAMP was analyzed using cAMP Direct Immunoassay Kit ab65355 (Abcam) accordingly to the producer instructions. Results were measured colorimetrically with plate reader (BioTek, Epoch, Winooski, VT, USA).

#### Protein kinases assay

Ganglions from two groups of cockroaches (control and exposed to 0.1 nM bendiocarb) were prepared as in the “Protein phosphorylation assay” section. Ganglions were placed in 500 µL of NP-40 lysis buffer and incubated for 15 min. Tissues were homogenized using glass homogenizer, and centrifuged (14,000 g, 30 min) afterwards. Supernatant was then used to perform direct immunoassays accordingly to the producer’s instruction: ab139437 for PKC activity and ab139435 for PKA activity (Abcam). Protein kinase level was measured colorimetrically on plate reader (BioTek, Epoch, Winooski, VT, USA).

#### LC–MS/MS analysis of octopamine level

Samples for LC–MS/MS were prepared from whole cockroaches, exposed beforehand to water (control) or 0.1 nM bendiocarb. Flowchart of experiments is presented in supplementary materials on Fig. SI1B. One minute or 1 h after exposure, insects were sacrificed by microwave irradiation (800 W, 30 s), weighted, and placed in the tube with 2 mL of water. Tissues were homogenized by ultrasound homogenizer (VCX-130) for 25 s and 666 µL of concentrated HCl was added to the homogenate. Samples were then centrifuged (for 10 min, at 14,000 g). Three hundred fifty microliters of supernatant was collected and 5 µL of d_3_-octopamine solution (internal standard, 1 µg/mL) and 26 µL of 20 M NaOH were added. Preliminary analytical conditions were developed using reference octopamine standard in a solution. The amount of octopamine was evaluated using LCMS-8045 tandem mass spectrometry (Shimadzu Corp.). Chromatographic separation was carried out on an Accucore™ Amide HILIC, 2.6 µm, 2.1 mm × 100 mm HPLC column. Twenty-five millimoles of ammonium formate with 0.05% formic acid (A) and 85% acetonitrile with 0.05% formic acid (v/v) (B) was used as the mobile phase. The separation was carried out in a linear gradient of 90–50% (v/v) acetonitrile for 4.5 min at a flow rate of 0.4 mL/min at 35 °C. In mass spectrometry, the samples were subjected to negative electrospray ionization (ESI) and ions were fragmented by collision-induced dissociation (CID). The ionization voltage was − 3 kV. Analysis of octopamine was based on multiple reactions monitoring (MRM transitions 136.1–92.3 and 136.1–65.2 for octopamine and 139.1–93.3 and 139.1–67.3 m/z for d_3_-octopamine).

### Fourth set of experiments—does exposure to 0.1 nM bendiocarb affect the effectiveness of high doses of bendiocarb?

#### Toxicity tests

In the following experiment, cockroaches were divided into 2 groups: control (Ctr) and exposed to 0.1 nM of bendiocarb (0.1 nM Bend). Flowchart of experiments is presented in supplementary materials on Fig. SI1A. Insects were exposed to bendiocarb as described in the “Insects’ exposure to bendiocarb” section. To evaluate the toxicological activity of 0.1 nM bendiocarb, we have performed the analysis of the level of insect paralysis. In this test, cockroaches were placed on their dorsal side by the experimenter and the time in which they were able to return to the ventral side was measured. The test was performed on a circular cork arena (50 cm of diameter) with a plastic wall around. The experiments were recorded with the video-camera (Logitech C920) to receive more precise measurement.

#### AChE activity assay

In the following experiment, cockroaches were divided into 2 groups: control (Ctr) and exposed to 0.1 nM of bendiocarb (0.1 nM Bend). The insects were exposed to bendiocarb as described in the “Insects’ exposure to bendiocarb” section. AChE activity was measured in ganglion homogenates using Ellman method (Ellman 1959). Shortly, homogenates were incubated with a desired concentration of bendiocarb (100 nM, 50 nM, 20 nM, 10 nM, 5 nM, 0.1 nM, and no bendiocarb (0 nM)) for 15 min. After that, time reaction was started with 0.1 mM acetylthiocholine and carried on for 30 min. Reaction was stopped by 2% SDS/1 mM DTNB. The level of the yellow product was measured at 410 nm on plate reader (BioTek, Epoch, Winooski, VT, USA). Protein level was determined using Bradford method.

### Data analysis

The analyses were made using ANOVA or Kruskal–Wallis tests for a few data with non-normal distribution. The differences between groups were tested by Mann–Whitney test. Significant differences in CO_2_ exchange between control and bendiocarb-treated groups were established using general linear model with treatment as a factor (bendiocarb or control) and cockroach mass as covariate followed by pairwise comparisons with Bonferroni correction. Statistical analyses were conducted in the IBM SPSS 25 Statistics software (IBM Corporation, Armonk, NY, USA). The results were expressed as mean values ± SE. The differences were considered significant when *p < 0.05, **p < 0.01, and ***p < 0.001. Inhibition curves were established using R software (R Core Team 2021) with “drc,” “sandwich,” and “lmtest” packages (Ritz et al. 2015; Zeileis 2004; Zeileis and Hothorn 2001).

## Results

### Does exposure to 0.1 nM bendiocarb change the behavior of insects?

**Fig. 1:**
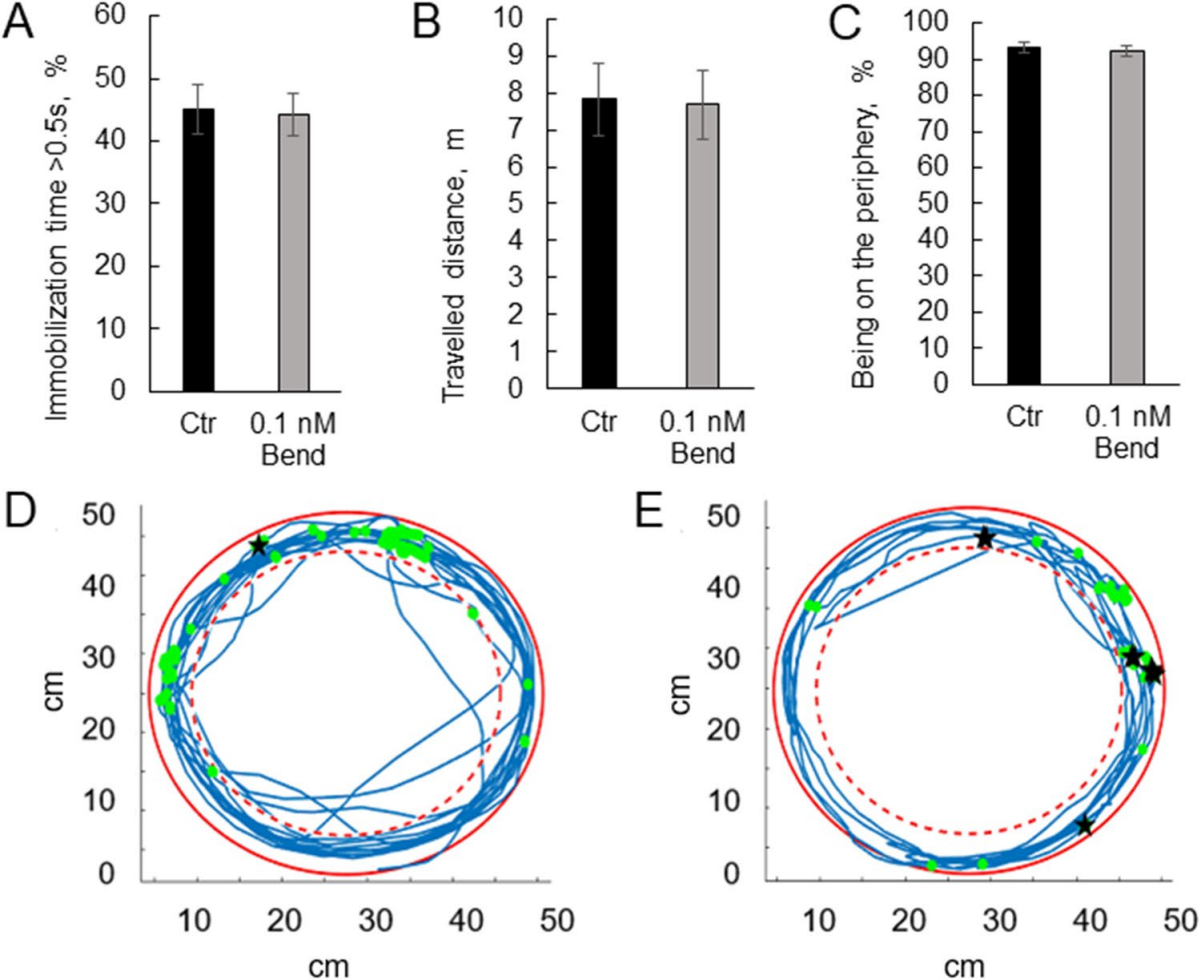
The motor activity of the cockroaches exposed to 0.1 nM bendiocarb. Black bars represent data for control insects (*n* = 21), while gray bars represent insects treated with 0.1 nM bendiocarb for 1 h (*n* = 21). **A** The percentage of time in immobility. **B** Distance travelled by insect in 10 min. **C** The percentage of time spent on periphery of arena. **D** Representative trace for control insect (blue lines) with indicated events of immobility longer than 0.5 s (green dots) and longer than 5 s (black stars). **E** Representative trace for insects treated with 0.1 nM bendiocarb

As bendiocarb-induced paralysis of cockroaches is due to the inhibition of acetylcholinesterase (Jankowska et al. 2019a, b), we used the motor activity test to determine how exposure of insects to very low bendiocarb concentration affects their mobility. The treatment with 0.1 nM bendiocarb did not change any of observed parameters (Fig. 1A–E). The percentage of immobility time was equal to 45.02 ± 4.01% in control and 44.21 ± 3.34% in 0.1 nM bendiocarb-treated group. Travelled distance was equal to 7.82 ± 0.96 m for control and 7.68 ± 0.9 m for 0.1 nM bendiocarb-treated insects, whereas percentage of time spent in periphery zone of arena was equal to 93.15 ± 1.45% and 92.10 ± 1.37% respectively.

**Fig. 2:**
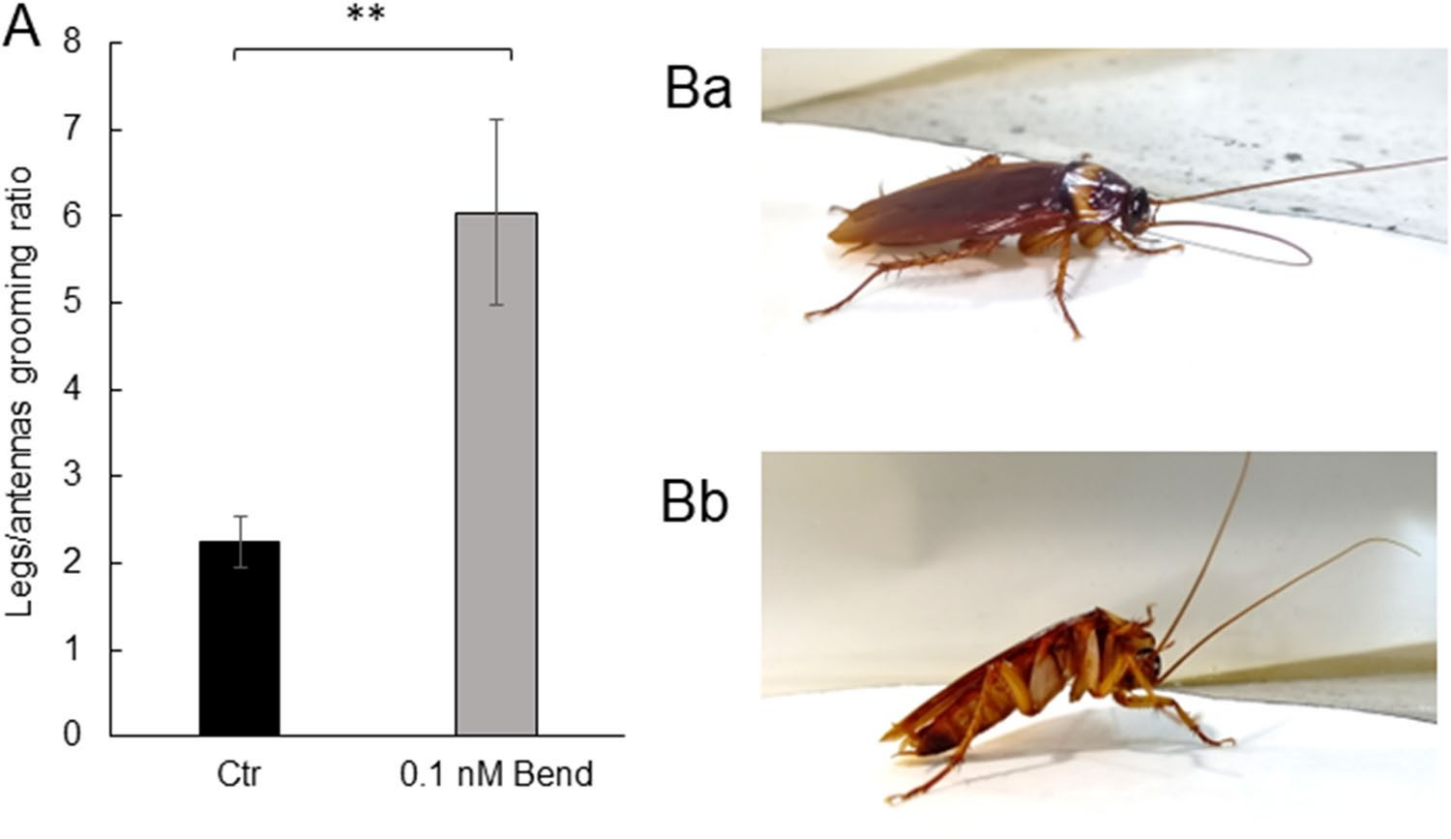
Grooming behavior. **A** Ratio of time of legs grooming to time of antennas grooming for control cockroaches (black bar, *n* = 30) and for insects exposed to 0.1 nM bendiocarb (gray bars, *n* = 28). **B** Cockroach grooming its antennas (Ba) and legs (Bb)

Because 0.1 nM bendiocarb did not cause any direct paralysis, we chose the test, in which we could observe a change in behavior as a response for the detection of chemical factors—grooming test. We have measured how long cockroaches groomed their legs in comparison to their antennas (Fig. 2A, B). For control insects, this ratio was equal to 2.25 ± 0.29, so the insects more frequently groomed their legs than antennas. In insects exposed to 0.1 nM bendiocarb, this ratio increased to 6.04 ± 1.07 (*p* = 0.007), which means that the insects groomed their legs around 3 times more often than the control ones.

**Fig. 3:**
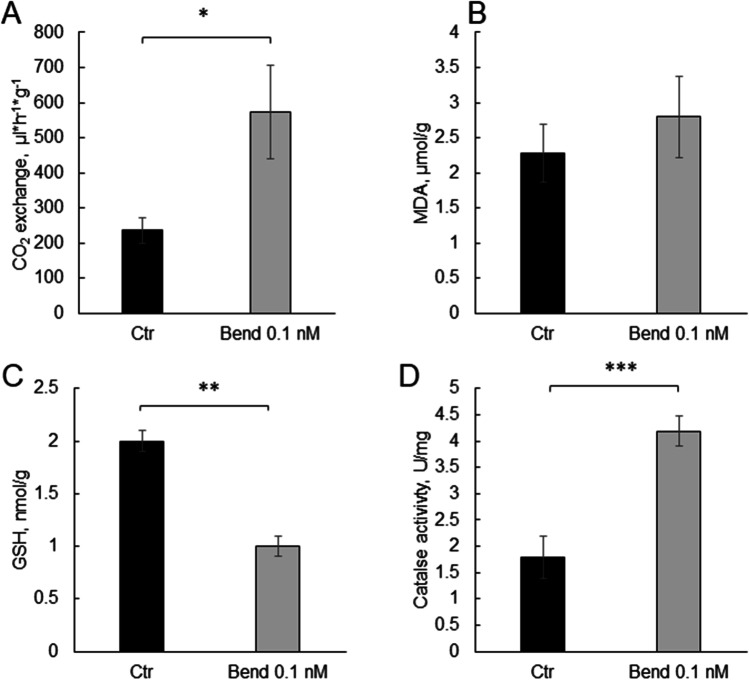
**A** CO_2_ exchange in cockroaches in control (black bars, *n* = 19) and after 1-h exposure to 0.1 nM bendiocarb (gray bars, *n* = 19). **B** MDA level, in control (black bars, *n* = 8) and after exposure to bendiocarb (gray bars, *n* = 8). **C** GSH level, in control (black bars, *n* = 8) and after exposure to bendiocarb (gray bars, *n* = 8). **D** Catalase activity in control (black bars, *n* = 8) and after exposure to bendiocarb (gray bars, *n* = 8)

Exposure to 0.1 nM bendiocarb did not affect the mobility of cockroaches, but insects were able to detect the insecticide. Thus, we took a closer look into physiological mechanisms that can be affected by the insecticide. First, we have evaluated metabolic rate of insects (Fig. 3A). Exposure to 0.1 nM bendiocarb resulted in a significant increase in CO_2_ release—from 235.1 ± 36.7 in control to 572.5 ± 133.0 µL·h^−1^·g^−1^ in bendiocarb-treated group (*p* = 0.016).

### Does exposure to 0.1 nM bendiocarb change the oxidative status in insects?

Then, we have analyzed the parameters of oxidative stress. The exposure to a very low dose of bendiocarb did not change the MDA levels significantly (*p* = 0.48, Fig. 3B). The level of antioxidant markers was significantly altered after the bendiocarb exposure. GSH level decreased 2 times (*p* = 0.004) and the catalase activity increased 2.3 times comparing to the control group (*p* < 0.001, Fig. 3C, D).

### Does 0.1 nM bendiocarb affect octopaminergic pathway?

**Fig. 4:**
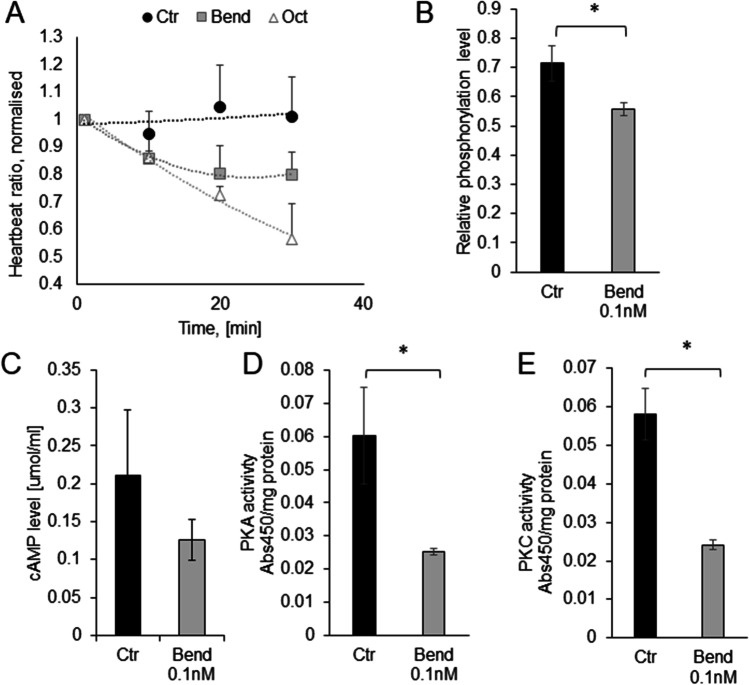
Exposure of insects to 0.1 nM bendiocarb changes the octopaminergic signaling pathway in the cockroach. Black bars correspond to the control preparation; gray bars correspond to the preparation from insects exposed to bendiocarb. **A** The heartbeat of the insect in control conditions (black dots, *n* = 7), after administration of 0.1 mM octopamine (white triangles, *n* = 7) and after administration of bendiocarb (gray squares, *n* = 7). **B** Relative phosphorylation level in ganglion tissue proteins, where 1 means fully phosphorylated, *n* = 4. **C** The level of cAMP in the preparation from ganglion tissue, *n* = 4. **D** Protein kinase A activity in the preparation from ganglions tissue, *n* = 5. **E** Protein kinase C activity in the preparation from ganglions tissue, *n* = 5

Significant changes in metabolism parameters prompted us to check, whether the exposure to trace amounts of bendiocarb resulted in the induction of stress response. We have chosen the physiological preparation that is very sensitive to octopamine, stress molecule of insects. First, we have evaluated the heartbeat of the cockroach. In this preparation, a high concentration of octopamine (0.1 mM) led to decrease in heartbeat (56.53 ± 12.81% of initial value 30 min after administration) in comparison to the control insects (100.88 ± 14.49% of initial values; *p* = 0.001; Fig. 4A). In the cockroaches treated with 0.1 nM bendiocarb, a significant decrease of the heartbeat was observed (80.03 ± 8.05% of initial value 30 min after administration).

**Fig. 5:**
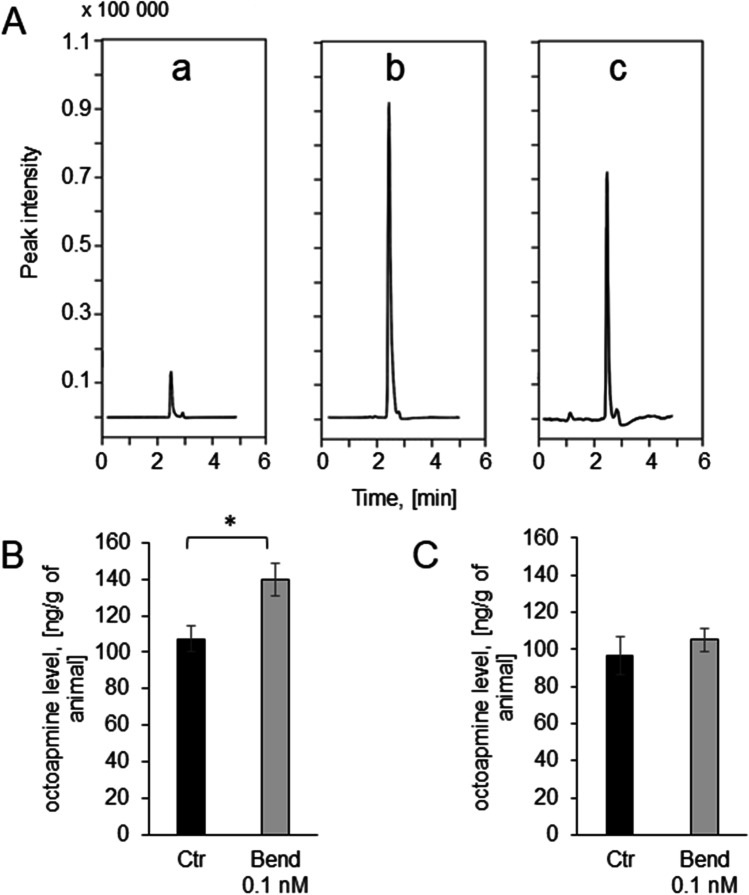
Exposure to 0.1 nM bendiocarb changes octopamine level in insects. **A** Chromatograms for (a) octopamine in the sample, (b) octopamine in the sample with addition of 200 ng/mL octopamine standard, and (c) deuterated octopamine standard. **B** The level of octopamine 1 min after exposure in the control group (black bar) and in the group exposed to 0.1 nM bendiocarb (gray bar), *n* = 18. **C** The level of octopamine 1 h after exposure in the control group (black bar) and in the group exposed to 0.1 nM bendiocarb (gray bar), *n* = 10

We also investigated the changes in octopamine signaling pathway evoked by 0.1 nM bendiocarb. The phosphorylation level of ganglion proteins decreased in the insects exposed to 0.1 nM bendiocarb (55.76 ± 2.09%; p = 0.03) in comparison to the value assessed in the control insects (71.38 ± 5.94%) (Fig. 5B). Cyclic AMP level also decreased from 0.21 ± 0.08 µmol/mL in the control insects to 0.12 ± 0.02 µmol/mL (Fig. 4C). Activities of both kinases were also lower in the insects exposed to bendiocarb. Control PKA activity was equal to 0.060 ± 0.014 Abs/mg and decreased to 0.025 ± 0.001 Abs/mg in the insects exposed to 0.1 nM bendiocarb (*p* = 0.02, Fig. 4D). Control PKC activity was equal to 0.058 ± 0.007 Abs/mg and decreased to 0.024 ± 0.001 Abs/mg in the insects exposed to 0.1 nM bendiocarb (*p* = 0.03; Fig. 4E).

Finally, we have examined whether the exposure to 0.1 nM bendiocarb raises octopamine level in insects, using sensitive LC–MS/MS method. We have observed the increase in octopamine level immediately after the exposure (from 107.29 ± 7.17 ng/g in the control group to 140.04 ± 8.93 ng/g in exposed group; *p* = 0.019; Fig. 5A, B). However, any changes in octopamine level were not visible 1 h after the exposure (Fig. 5C).

### Does exposure to 0.1 nM bendiocarb affect the effectiveness of high doses of bendiocarb?

Considering the possible impact of trace amounts of insecticides on public health, we have evaluated how the exposure to 0.1 nM bendiocarb changes the sensitivity of the animal to an effective dose of insecticide. We chose the concentration of 1 µM of bendiocarb, since we know this is breakpoint concentration, where effects of its use are visible (Jankowska et al. 2019a).

**Fig. 6:**
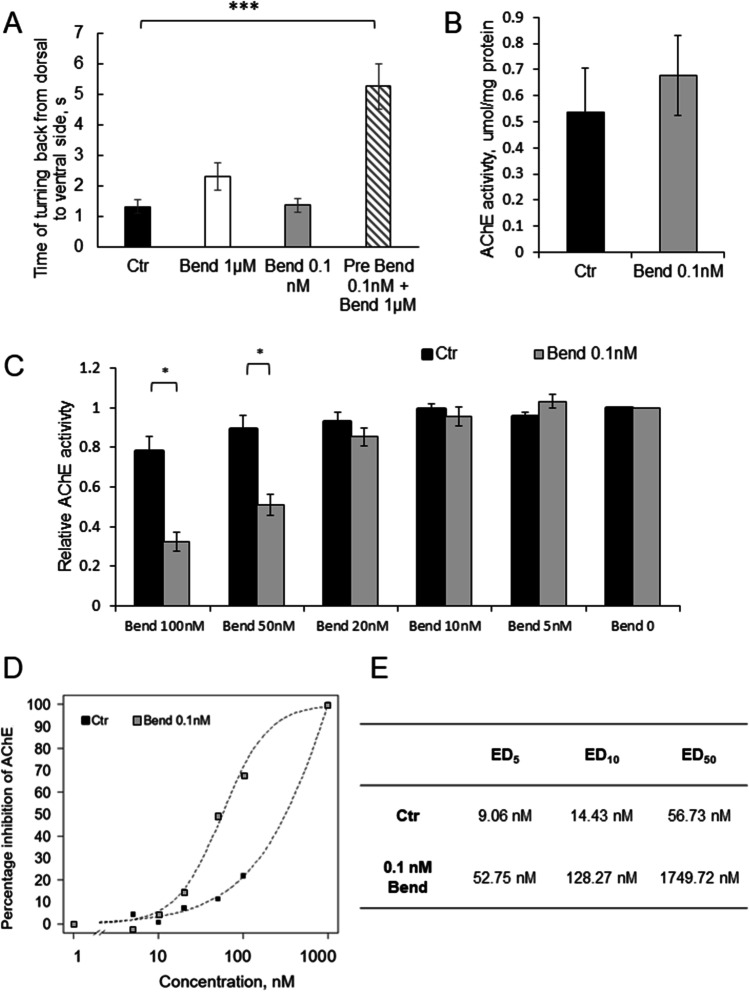
Trace amounts of bendiocarb change sensitivity to effective doses of insecticides. **A** Motor skills of the control insects (black bar, *n* = 28), the insects exposed to 1 µM bendiocarb (white dark bar, *n* = 47), the ones exposed to 0.1 nM bendiocarb (gray bar, *n* = 24), and the insects preincubated with 0.1 nM bendiocarb and then exposed to 1 µM bendiocarb (hatched bar, *n* = 46). **B** Original activity of AChE from ganglions of the control insects (black bar) and the insects exposed to 0.1 nM bendiocarb (gray bar). **C** Relative activity of AChE from ganglions of the control insects (black bars) and the insects exposed to 0.1 nM bendiocarb (gray bars). AChE preparation from ganglions was incubated with a range of bendiocarb concentrations. **D** Inhibition curve for bendiocarb in the control preparation (black squares) and in the preparation of insects exposed to 0.1 nM bendiocarb (gray squares). **E** Calculated ED_5_, ED_10_, and ED_50_ values for bendiocarb in the preparation from control insects and insects exposed to 0.1 nM bendiocarb

In motor skill test, we have observed the ability of insects to turn back from dorsal to ventral side (Fig. 6A). In control conditions, insects were able to turn back in 1.32 ± 0.22 s. Insect incubation with bendiocarb in concentration of 1 µM slightly increased the time of turning back to 2.32 ± 0.46 s, while insect incubation with 0.1 nM bendiocarb did not cause any effect—the time of turning back remains on the level of 1.37 ± 0.23 s. When we preincubated insects with 0.1 nM bendiocarb and after that applied final 1 µM bendiocarb, the observed time of turning back increased substantially to 5.28 ± 0.74 s (*p* < 0.001).

We have also evaluated the level of inhibition of AChE enzyme by bendiocarb in the preparation from the insects previously exposed to 0.1 nM bendiocarb and from the control insects. In the insects exposed to 0.1 nM bendiocarb for 1 h, the level of AChE activity in ganglion was slightly higher (0.67 ± 0.15 µg/mg protein) than in the control insects (0.53 ± 0.17 µg/mg protein); thus, no enzyme inhibition was observed (Fig. 6B). When we incubated homogenates with bendiocarb in the range of concentrations, the inhibition of the enzyme was more intensive in the insects previously exposed to 0.1 nM bendiocarb concentrations, e.g., 100 nM bendiocarb caused 21.89% inhibition of enzyme in control preparations, while in the insects pre-exposed to 0.1 nM concentrations of bendiocarb, the inhibition was equal to 67.74% (Fig. 6C). Inhibition curve estimated for the preparation from the control insects and the preparation from the insects exposed to 0.1 nM bendiocarb is clearly moved into the direction of lower concentrations of bendiocarb (Fig. 6D), which is also represent by lower ED (effective dose) values (Fig. 6E).

## Discussion

In the recent years, more and more attention is focused on the effects of low concentrations of toxic substances that do not cause direct effects. Continuous exposure to trace amount of insecticides can be harmful for all organisms. In our study, we have investigated the effects of exposure of cockroaches (*Periplaneta americana*) to bendiocarb, carbamate insecticide, in a concentration lower than studied anywhere else—0.1 nM.

Such an extremely low concentration of the insecticide did not induce any paralyzing effect. However, in our study, we observed changes in grooming pattern; thus, it means that cockroaches are able to detect trace amounts of bendiocarb. Grooming in insects is a typical behavior, which purpose is to clean sensillum areas from environmental pollutions and cuticular secretion (Böröczky et al. 2013). Antenna cleaning is the major reaction that occurs after contact with odor substances. In the case of our study, the control insects groomed their antennas over twice more often than their legs. A similar pattern of grooming in control insects was observed previously (Zhukovskaya 2014). Changes in two aminergic systems were shown to alter the pattern of grooming: activation of dopaminergic system led to the equalization of the level of antennas and legs grooming (Weisel-Eichler et al. 1995), whereas the activation of octopaminergic system led to the increase in leg grooming considerably (Carrazoni et al. 2016). In our study, cockroaches after contact with 0.1 nM bendiocarb more frequently groomed their legs than the control ones, which can suggest the activation of octopaminergic response to the detected substances.

Trace amounts of insecticides can induce changes in neural and muscular tissues leading to disturbances in physiological processes. To characterize physiological state of an insect, metabolic rate and respiratory patterns are commonly assessed. In our experiments, we have shown that 0.1 nM bendiocarb increased the respiration rate of cockroaches. Kestler (1991) showed that sublethal doses of pesticides induce changes in *Periplaneta americana* respiratory pattern, which is a sensitive indicator of stress. The same observation was confirmed in Woodman et al. (2008) experiments. Low concentration of phosphine (800 ppm; 2-h exposure), which did not cause any mortality or sublethal effects, immediately disrupted normal gas exchange pattern in *P. americana*. The consequence of sublethal insecticide doses can be the modification of physiological processes in insects.

Changes in respiratory pattern are connected with oxidative stress in insects (Hetz and Bradley 2005). In addition, xenobiotic exposure affects the level of oxidative stress and antioxidant status in insects (Aslanturk et al. 2011). Therefore, we decided to assess the effect of exposure to trace amount of bendiocarb on the oxidant/antioxidant status.

Malondialdehyde (MDA) is a known marker of oxidative stress, being a major product of lipid peroxidation by reactive oxygen species. MDA levels increase after contact with pesticides. Treatment with LC_50_ concentration of methidation, organophosphate pesticide, induced an increase in MDA level in midgut tissues 48 h after exposure in *Gypsy moth* (Aslanturk et al. 2011). In our study, only 1-h exposure to sublethal doses of bendiocarb was sufficient to induce a tendency to increase in MDA level. This suggests an increase in oxidative stress, especially when we compare it to the changes in antioxidant levels—catalase and glutathione.

An antioxidant enzyme, such as catalase, is an important component of organism defense against oxidative stress. Increased level of catalase is therefore an indicator of an organisms’ attempt to cope with environmental stressors, such as pesticides. Corresponding to our results, the increase in CAT activity was observed in honey bees from areas of intensive agriculture (Chakrabarti et al. 2015).

The reduction in GSH concentration, observed in our experiments, was probably the result of a direct utilization of GSH as an antioxidant in elimination of reactive oxygen species elevated by the bendiocarb exposure. The exposure of cockroaches to 0.1 nM bendiocarb induced a decrease in GSH levels which was accompanied by an increase in CAT activity. This indicates an adaptive response that can help to cope with insecticide intoxication. GSH together with CAT are effective reactive oxygen species scavengers and are involved in protection against lipid peroxidation due to pesticide exposure, even when it is applied in extremely low doses.

We have shown that low doses of bendiocarb also decreased the heartbeat of the cockroach. The preparation of insect’s heart is commonly known to be sensitive to octopamine (Roeder 2020). In our study, 0.1 mM octopamine reduced the heartbeat rate, which was also observed in bath application of octopamine in *Crassostrea virginica* (Hoque et al. 2013). It was evidenced that octopamine in high concentration can “antagonize” octopamine receptors (Papaefthimiou and Theophilidis 2011). The similar modes of action on heart preparation of low doses of bendiocarb and high dose of octopamine suggest that application of bendiocarb can lead to an elevation of octopamine level. As a result, we can observe the effect of inhibition of octopamine receptors and decrease in heartbeat. These results indicate that octopamine may be involved in the insect response to a very low dose of bendiocarb.

In the next part of our research, we observed an indirect effect of 0.1 nM bendiocarb on the elements of the octopamine receptors cellular signaling pathway. Activation of octopamine receptors can increase intracellular calcium level or cAMP level, which activates protein kinase C or protein kinase A respectively (Farooqui 2012). In our study, we observed a decline of all parameters related to the cellular octopamine pathway, which can suggest that bendiocarb exposure led to octopamine elevation to the level which blocked octopamine receptors. Further research is necessary to evaluate which type of octopamine receptors is involved in sublethal bendiocarb action.

In the last step of our research, we have shown that preincubation with 0.1 nM bendiocarb changes insect susceptibility for the higher doses of the substance, both in toxicological studies and in biochemical AChE activity evaluation. Similar mechanism was previously shown by us for menthol, which potentiated bendiocarb efficacy through octopaminergic system (Jankowska et al. 2019a, b). Also, other factors which change phosphorylation activity of kinases were shown to increase or decrease AChE sensitivity for carbamate insecticides (Abd-Ella et al. 2015). It should be stressed that substances such as bendiocarb are neurotoxins not only for insects, but also for mammals, and there is possibility that similar increase in toxicity in organism exposed to trace amounts of insecticides will be observed in different organism, including humans.

Effects of sublethal doses of bendiocarb on various organisms were tested before (Gazsi et al. 2021; Kunkel et al. 2001; MacKenzie and Winston 1989; Tarek et al. 2018). However, in all available researches, the tested concentrations were only a little bit lower than LD_50_ and admittedly the lethal effect was not observed, but evident physiological and behavioral changes were present. In two available researches (Campero et al. 2007; He et al. 2013), no physiological changes were observed, when other carbamate insecticide—carbaryl—was applied in 10 µg/L concentration. In our study 500 × lower concentration was used. It is possible that here we can observe effect of hormesis (Cutler 2013). High concentrations of bendiocarb cause direct effects on the enzyme AChE, thus leading to death or paralysis. Intermediate concentrations (as 10 µg/L) do not cause direct effects, but are high enough to evoke defense mechanism, leading to detoxification. It is possible that extremely low insecticide concentrations are insufficient to trigger the organism’s defenses, but are still able to influence the insect physiology. Continuous exposure to extremely low insecticide concentrations should then be examined to find out its impact on the organisms.

## Conclusion

Exposure to extremely low concentration (0.1 nM) of insecticide—bendiocarb—significantly changed the insects’ behavior and physiology. As a result, the insects’ susceptibility for effective doses of the same insecticide was increased. Similar effects on other organisms, including humans, cannot be excluded and therefore the effects of trace pesticide residues should be considered in the public health risk assessment.

## Supplementary Information

Below is the link to the electronic supplementary material.

Supplementary file1 Fig. SI1 The course of experiments. A) The insectmaintenance, exposure to 0.1 nM bendiocarb and examination used in allexperiments with exception of LC/MS and heartbeat analysis. B) The schematiccourse of LC/MS experiment. C) The schematic course of heartbeat analysis(details in text). Created with BioRender.com (PNG 1807 KB)

## Data Availability

The datasets generated during the current study are available in the repository: https://repod.icm.edu.pl/dataset.xhtml?persistentId=doi%3A10.18150%2FE77PMC&version=DRAFT.
