# Supplementary figures and images for: Sublethal biochemical, behavioral, and physiological toxicity of extremely low dose of bendiocarb insecticide in Periplaneta americana (Blattodea: Blattidae)
Source: Environ Sci Pollut Res Int. 2023 Feb 6;30(16):47742–54. doi: 10.1007/s11356-023-25602-8 (PMC10097796; doi:10.1007/s11356-023-25602-8)

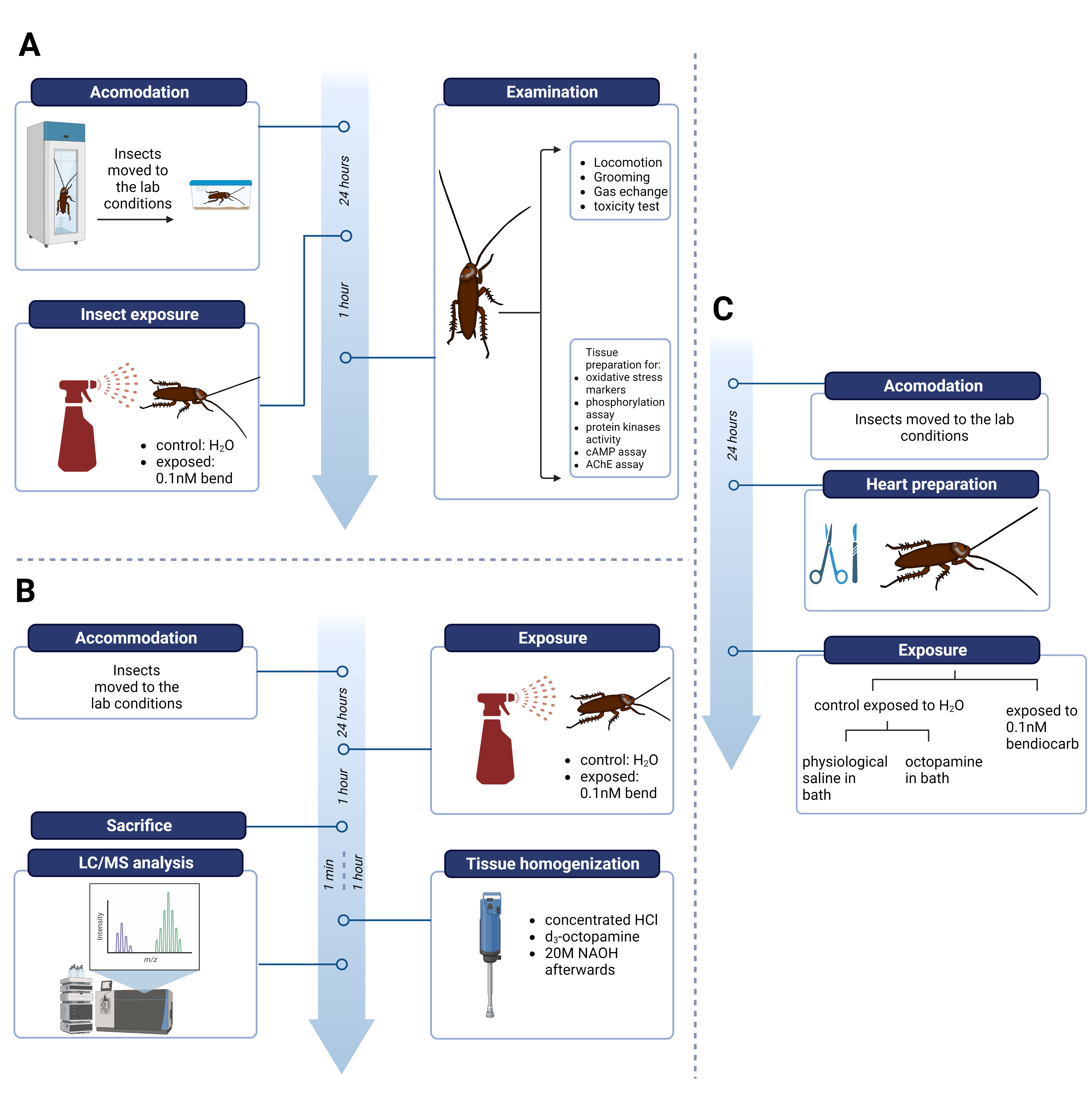

Supplement: Supplementary file 1 — Supplementary file1 Fig. SI1 The course of experiments. A) The insectmaintenance, exposure to 0.1 nM bendiocarb and examination used in allexperiments with exception of LC/MS and heartbeat analysis. B) The schematiccourse of LC/MS experiment. C) The schematic course of heartbeat analysis(details in text). Created with BioRender.com (PNG 1807 KB) [file 11356_2023_25602_MOESM1_ESM.png]
